# Discovery and validation of Hsa-microRNA-3665 promoter methylation as a potential biomarker for the prognosis of esophageal squaous cell carcinoma

**DOI:** 10.1007/s10147-024-02656-3

**Published:** 2024-12-04

**Authors:** Jinsong Zhou, Shuang Liu, Juwei Zhang, Qiaoyan Zeng, Zheng Lin, Rong Fu, Yulan Lin, Zhijian Hu

**Affiliations:** 1 Department of Epidemiology and Health Statistics, School of Public Health, Fujian Medical University, 1 Xue Yuan Road, University Town, Fuzhou, 350122 China; 2 Sun Yat-Sen University Cancer Center/Cancer Hospital, Guangzhou, 510060 China; 3 Key Laboratory of Ministry of Education for Gastrointestinal Cancer, Fujian Medical University, Fuzhou, 350108 China

**Keywords:** Esophageal squamous cell carcinoma, Hsa-miR-3665, Promoter methylation, Prognosis

## Abstract

**Background:**

Methylation of microRNA (miRNA) promoters associated with diseases is a common epigenetic mechanism in the development of various human cancers. However, its relationship with prognosis in esophageal squamous cell carcinoma (ESCC) remains unclear. This study aims to explore the association between the methylation level of has-miR-3665 promoter and prognosis in ESCC.

**Methods:**

Human miRNA data were downloaded from miRbase, and we identified CpG islands of these human miRNAs by genomics browser analysis. MiRNA methylation levels were detected by methylation-specific high-resolution melting. Gene ontology (GO), and Kyoto Encyclopedia of Genes and Genomes (KEGG) analyses were used to explore the molecular mechanism of hsa-miR-3665. Cox regression analysis was used to investigate prognostic factors. The overall survival rate was predicted by a nomogram.

**Results:**

We found that 88 human miRNAs had promoter methylatio, of which 15 miRNAs were found to be epigenetically regulated in ESCC cells compared with their normal counterparts, including hsa-miR-3665. Meanwhile, hsa-miR-3665 expression was significantly lower in ESCC tumour tissue than in adjacent tissue (*P* = 0.03). GO and KEGG analyses demonstrated that the target genes are involved in protein transport, transcription regulator activity, MAPK and RAS signaling pathway. High hsa-miR-3665 promoter methylation levels were associated with a poor prognosis (*HR* = 3.89, 95% *CI* 1.11 ~ 13.55). Moreover, a nomogram incorporating the hsa-miR-3665 methylation level and clinical factors presented a good performance for predicting survival in the training and validation tests, with C-indices of 0.748 and 0.751, respectively.

**Conclusions:**

High hsa-miR-3665 promoter methylation levels may be a potential biomarker for the progression of ESCC.

**Supplementary Information:**

The online version contains supplementary material available at 10.1007/s10147-024-02656-3.

## Background

Esophageal cancer, a global public health problem, is one of the most common digestive system cancers around the world [1, 2]. Esophageal squamous cell carcinoma (ESCC) is the predominant type of esophageal cancer in China [3]. Although the available treatment has improved recently, the prognosis of ESCC remains unsatisfactory [4]. An increasing number of studies evaluating miRNA expression in regard to ESCC prognosis have been reported [5, 6]; however, only a few studies have explored the association between miRNA promoter methylation and ESCC prognosis.

DNA methylation is a well-established epigenetic mechanism critical for cancer, and it mainly occurs in CpG islands [7]. It has been considered a potential biomarker for cancers [8, 9]. MiRNAs are the most widely described ncRNAs, and researchers have shown that miRNAs could play significant roles in many biological processes, including cellular development, differentiation, invasion, etc. [10, 11]. Studies have reported that miRNA genes have CpG islands in their promoter regions, which could be affected by methylation [12]. In fact, approximately 20% of all miRNAs are embedded within CpG islands [13]. In recent studies, it was revealed that epigenetic alterations were associated with the dysregulation of miRNAs in cancers. For instance, Gao et al. [14] pointed out that miR-145-5p promoter hypermethylation inhibited miR-145-5p expression through epigenetic inactivation and low miR-145-5p expression was associated with a worse prognosis in laryngeal squamous cell carcinoma. Similarly, in prostate cancer, significantly higher miR-34 methylation levels independently predicted a shorter disease-free survival [15]. These studies demonstrated that miRNA promoter methylation is a significant predictor of patient outcomes. For ESCC, there are two studies indicated hypermethylation-mediated inactivation of miR-124, miR-203a and miR-203b may be useful for a poor prognostic marker for ESCC patients [12, 16]. However, these studies did not systematically screen for human miRNA promoter methylation and focused only on specific miRNAs promoter methylation. Hence, studies comprehensively exploring miRNA promoter methylation profiles in regards to the ESCC prognosis are necessary.

MiR-3665 was initially recognized for its role in neurodevelopment, where it influences neurodevelopmental injuries and cognitive disorders [17, 18]. It is also linked to aging and rejuvenation [19]. Research has demonstrated that miR-3665 influences immune system function in lung cancer by targeting the COL1A1 and COL5A1 genes [20]. Furthermore, in endometrial cancer, miR-3665 is downregulated due to promoter hypermethylation [21]. However, the role of miR-3665 promoter methylation in ESCC remains unclear. Elucidating the relationship between miR-3665 and prognosis in ESCC could help identify a new potential biomarker.

In this study, we downloaded human miRNA data from miRbase and identified the CpG islands of these human miRNAs using several genomics browser analyses. Furthermore, eighty-eight miRNA promoter methylation sites were verified in ESCC cell lines by using miProfileTM miRNA qPCR microarray technology. Then, the ESCC tumour tissue miRNA methylation level was detected by methylation-specific high-resolution melting. Here, we found that high hsa-miR-3665 promoter methylation levels in ESCC patients were significantly correlated with a worse prognosis. The hsa-miR-3665 promoter methylation levels may be potentially useful as a novel biomarker for ESCC prognosis.

## Methods

### Human miRNAs sources download

The hairpin and mature sequences of miRNA were identified in the miRBase database. In addition, all sequence and annotation data are also available for download. We downloaded the human miRNA data through miRBase (http://www.mirbase.org/, version 18.0).

### The CpG islands of miRNAs analysis

We identified CpG islands of these human miRNAs using several genomics browser analyses, such as the UCSC Genome Browser (http://genome.ucsc.edu/), CpG Island Searcher (http://www.cpgislands.com/), and the European Bioinformatics Institute (EBI, http://www.ebi.ac.uk/Tools/emboss/cpgplot/). In this study, the threshold criteria for CpG islands of miRNAs were as follows: (1) UCSC Genome Browser: GC% ≥ 50%, length > 200 bp, Obs/Exp CpG > 0.6. (2) CpG Island Searcher: GC% = 60%, ObsCpG/ExpCpG = 0.7, length = 300 bp, gap between adjacent islands = 100 bp. (3) EBI: Obs/Exp = 0.7, MinPC = 60, Length = 300. We excluded miRNAs located on the X chromosome, and for two miRNAs with similar positions, we chose miRNAs with a large ObsCpG/ExpCpG value. To improve the accuracy of the CpG islands of prediction, we chose an intersection among the three databases.

### Cell culture

Het-1A, Eca109, TE-1, CaES-17 and EC9706 cells were obtained from the Shanghai Institute of Biochemistry and Cell Biology and were cultured in Dulbecco's modified Eagle's medium (Sigma, USA) supplemented with 10% foetal bovine serum (FBS) (HyClone, USA). The cells were cultured at 37 °C in a humidified 5% CO2 incubator (Thermo, USA).

### Cell treatment

To estimate the role of epigenetic mechanisms in miRNAs, 5-aza-2'-deoxycytidine (DAC) was used as follows. Two groups were created: ESCC cell lines vs. the Het-1A cell line and ESCC cell lines with 5-aza-2ʹ-deoxycytidine vs. ESCC cell lines without 5-aza-2 ʹ -deoxycytidine. The cells were treated with 1 μM 5-aza-dC for 72 h. After incubation, the cell miRNAs were isolated using the Total TRIzol Reagent Kit (Life Technologies, America). The relative quantifications of the miRNAs between the ESCC and Het-1A cell lines and between the ESCC cell lines with DAC and without DAC were detected by real-time RT-PCR (qPCR), and miProfileTM miRNA qPCR microarray technology was used. U6, U44, U48 and U47 snRNAs were used as internal controls, and the premier sequences of the miRNAs and controls are presented in Table S1. Additionally, we used a reverse transcription control, a positive PCR control, and a no template control. miRNAs with fold changes (ESCC cell lines vs. Het-1A cell line) < 0.5 and fold changes (ESCC cell lines with DAC vs. ESCC cell lines without DAC) > 2 were screened.

### Clinical samples and follow-up

We conducted a study of patients newly diagnosed with ESCC at the Zhangzhou Affiliated Hospital of Fujian Medical University in Fujian, China, from December 1, 2012, to August 31, 2014. Participants who underwent surgery at Zhangzhou Affiliated Hospital of Fujian Medical University were recruited. The diagnosis was confirmed by two pathologists. Patients with other cancers were excluded. Before the patients underwent surgical excision, they did not receive chemotherapy or radiotherapy. A total of 145 ESCC tissues were collected. This study was approved by the Institutional Review Boards of Fujian Medical University (NO. 2,011,052), and all participants signed informed consent forms.

### MiRNAs extraction

MiRNAs were extracted from cells using the Total TRIzol Reagent Kit (Life Technologies, America). A NanoDrop ND-1000 system (NanoDrop, Thermo, America) was used to detect the concentration of all RNA samples.

### Quantitative real-time PCR

To validate the miRNA expression levels, qPCR was performed using an SYBR kit (Takara, Japan) on a 7500 PCR System (Applied Biosystems, Thermo, America). The premier for the candidate miRNAs and internal controls were purchased from Guangzhou Funeng (Guangzhou, China). Table S1 shows the premier sequences. The 2^−ΔΔCT^ method was applied to calculate the relative expression values for the target miRNA, which were normalized to the internal control.

### Methylation-specific high-resolution melting (MS-HRM) analyses

Genomic DNA was extracted from ESCC tissue specimens using an adsorption column. Tissue DNA was treated with bisulfite using an EpiTect PLUS DNA Bisulfite kit (QiaGen, Germany) according to the manufacturer’s protocol. Methylation-sensitive high-resolution melting (MS-HRM) assays were used to detect the methylation levels. Briefly, bisulfite-modified unmethylated and methylated standard DNA (Qiagen GmbH) was mixed, giving the samples 0, 25, 50, 75, and 100% methylation degrees for calibration. A standard curve with known methylation degrees was included in each run. The hsa-miR-3665 premier sequence is listed in Table S2. The hsa-miR-3665 amplification conditions were 39 cycles at 95 °C for 30 s, 56.7 °C for 30 s and 72 °C for 15 s. High-resolution melting analysis with LightScanner TM was used to analyse the products after PCR. The HRM data were calculated using High-Resolution Melting Software version 2.0.1 (Applied Biosystems). We divided 145 ESCC patients into two groups according to their 50% methylation levels.

### Prediction of target genes of hsa-miR-3665

The target genes of hsa-miR-3665 were predicted using the following miRPathDB (https://mpd.bioinf.uni-sb.de/overview.html), miRWalk (http://mirwalk.umm.uni-heidelberg.de/) and TargetScan (http://www.targetscan.org/index.html). The intersection of these three software results was used as the final target gene of hsa-miR-3665.

### GO and KEGG pathway enrichment analysis of target genes

To analyze the functions of the predicted target genes of hsa-miR-3665, Gene Ontology (GO) (http://www.geneontology.org) was used for gene functional enrichment of predicted target genes. Kyoto Encyclopedia of Genes and Genomes (KEGG) data (http://www.kegg.jp/kegg/) was used for analyzing the roles of target genes. The human genome was used as a genetic background. *P* value < 0.05 was considered statistically significant in GO terms and KEGG pathways.

### Statistical analysis

The paired sample t-test was applied to evaluate the miRNA expression level between tumour tissue and the corresponding adjacent tissues. The association between the methylation ratio of miRNAs and the clinical factors was assessed using Chi-square test and multivariate logistic regression. Cox proportional hazard regression was used to investigate the prognostic factors for ESCC. All of the participants were randomly divided into either the training or validation set (the split ratio was 2:1), with the training and validation sets being used to establish the predictive model and to construct the nomogram [22]. The C-indices and calibration curves were created to determine whether the predicted survival and actual survival were in concordance. All statistical analyses and visualizations were performed using R software (version 4.1.2). Differences were identified as considered statistically significant for two-sided *P* values < 0.05.

## Results

### Screening the CpG islands of miRNAs

**Fig. 1:**
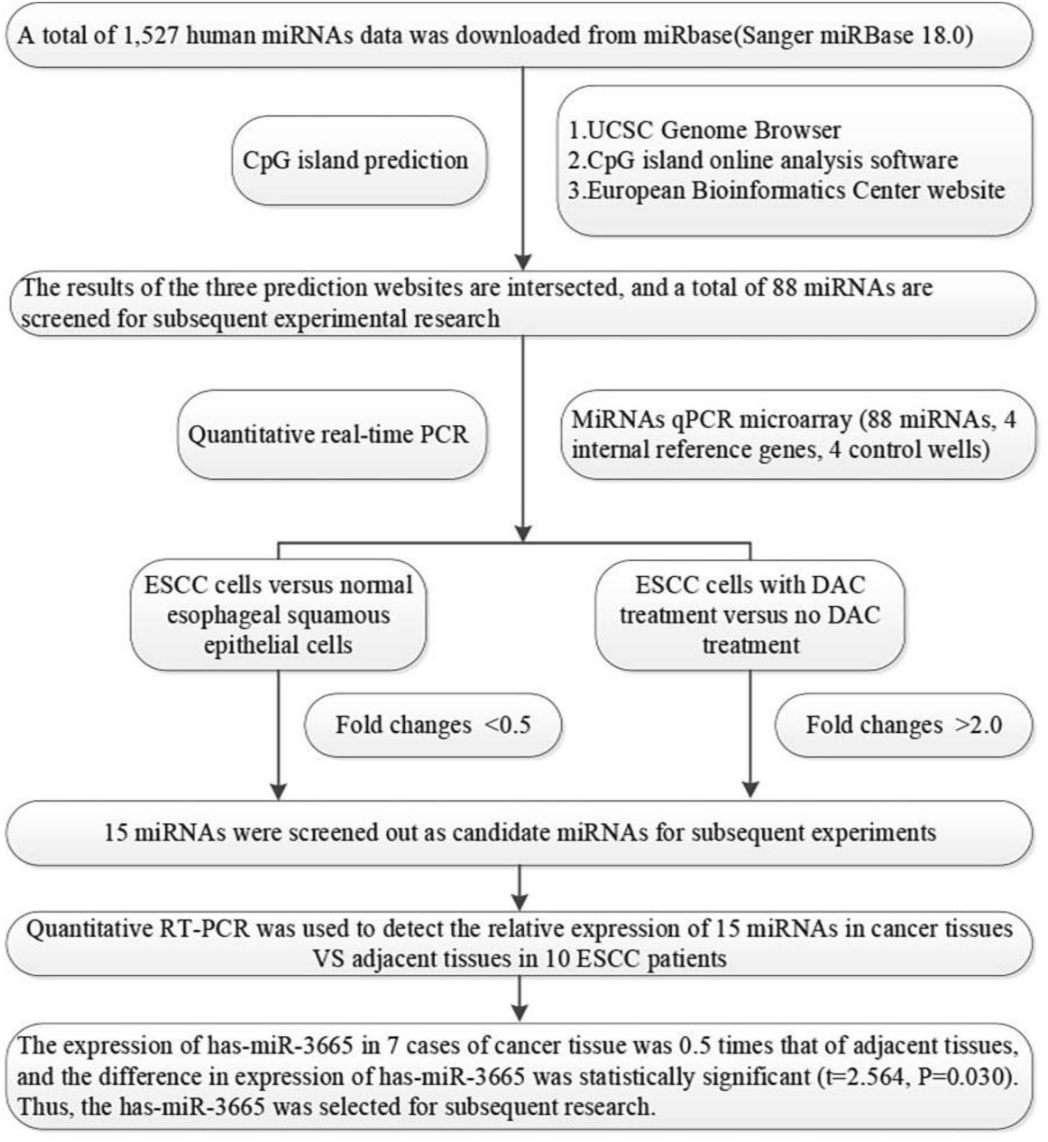
The flowchart of screening 15 miRNAs from 1527 miRNAs

In the preliminary screening, a total of 1,527 human miRNA datasets were downloaded from miRbase. The CpG islands of 1,527 miRNAs were predicted by bioinformatics methods, including the UCSC Genome Browser, CpG Island Searcher and European Bioinformatics Institute website. In this study, 88 miRNA promoter methylations were selected at a common intersection among the three databases (Table S3). We also used the miProfileTM miRNA qPCR microarray of ESCC cell lines (tumour cell lines vs. the Het-1A cell line; tumour cell lines with DAC vs. tumour cell lines without DAC). Screening of candidate miRNAs was conducted according to the relative quantification (RQ) of the ESCC cells with and without DAC treatment > 2 times and the RQ of ESCC cells and normal esophageal squamous epithelial cells < 0.5 times and the results showed that 15 miRNAs might have differential promoter methylation in ESCC (Table 1). The flowchart of screening the 15 miRNAs from the 1527 miRNAs is presented in Fig. 1.

**Table 1:** Candidate miRNAs screened in ESCC cell lines

No	miRNAs	RQ(CC vs NC)*	RQ(DAC vs DMSO)*
1	hsa-miR-9-5p	0.005	17.563
2	hsa-miR-203	0.128	3.535
3	hsa-miR-375	0.182	2.010
4	hsa-miR-1258	0.449	2.458
5	hsa-miR-1914-5p	0.116	2.656
6	hsa-miR-3665	0.206	2.896
7	hsa-miR-4470	0.251	3.215
8	hsa-miR-4479	0.107	3.411
9	hsa-miR-4530	0.158	3.383
10	hsa-miR-4634	0.467	3.137
11	hsa-miR-4664-5p	0.220	7.804
12	hsa-miR-4665-5p	0.284	7.364
13	hsa-miR-4674	0.356	3.342
14	hsa-miR-4687-5p	0.378	3.434
15	hsa-miR-4734	0.236	2.629

*RQ (CC vs NC) refers to the relative expression of ESCC cells relative to normal esophageal squamous epithelial cells (Het-1A cell line); RQ (DAC vs DMSO) refers to the relative expression of ESCC cells with DAC treatment versus no DAC treatment

### The expression of the fifteen miRNAs in ESCC tissue samples

The expression levels of 15 miRNAs in ESCC were detected by qPCR. First, ten ESCC tissues were used to perform the initial verification. The relative quantification of the fifteen miRNAs between the ESCC tissues and the adjacent tissues in ten patients with ESCC was compared. The expression of the 15 miRNAs is shown in Table S4. The number of patients whose miRNA expression in ESCC tissues was less than 0.5 times that of adjacent tissues was: hsa-miR-9-5p (4 cases), hsa-miR-203 (5 cases), hsa-miR-375 (6 cases), hsa-miR-1258 (2 cases), hsa-miR-1914-5p (3 cases), hsa-miR-3665 (7 cases), hsa-miR-4470 (2 cases), hsa-miR-4479 (3 cases), hsa-miR-4530 (4 cases), hsa-miR-4634 (5 cases), hsa-miR-4664-5p (3 cases), hsa-miR-4665-5p (4 cases), hsa-miR-4674 (3 cases), hsa-miR-4687-5p (5 cases) and hsa-miR-4734 (4 cases). The relative quantification of hsa-miR-3665 was significant (*t* = 2.564, *P* = 0.030) (Table 2). Besides, there was an association between the expression level and promoter methylation status of hsa-miR-3665 in ten ESCC (*P* < 0.05, Table S5). Thus, we chose hsa-miR-3665 for further research.

**Table 2:** Comparison of miRNAs expression in ESCC tissues and adjacent tissues

miRNAs	Normality test		Paired sample *t* test	
	*Z*	*P*	*t*	*P*
hsa-miR-9-5p	0.608	0.853	1.212	0.256
hsa-miR-203	0.576	0.895	− 0.040	0.969
hsa-miR-375	0.458	0.985	1.361	0.207
hsa-miR-1258	0.744	0.637	0.758	0.468
hsa-miR-1914-5p	0.587	0.880	0.931	0.376
hsa-miR-3665	0.381	0.999	2.564	0.030
hsa-miR-4470	0.671	0.759	− 0.182	0.860
hsa-miR-4479	0.508	0.959	0.659	0.526
hsa-miR-4530	0.597	0.868	1.369	0.204
hsa-miR-4634	0.549	0.924	1.866	0.095
hsa-miR-4664-5p	0.476	0.977	0.063	0.951
hsa-miR-4665-5p	0.522	0.948	0.977	0.354
hsa-miR-4674	0.530	0.941	0.266	0.796
hsa-miR-4687-5p	0.427	0.993	1.339	0.214
hsa-miR-4734	0.376	0.999	− 0.165	0.873

### Target prediction of hsa-miR-3665 and GO and KEGG pathway enrichment analysis

**Fig. 2:**
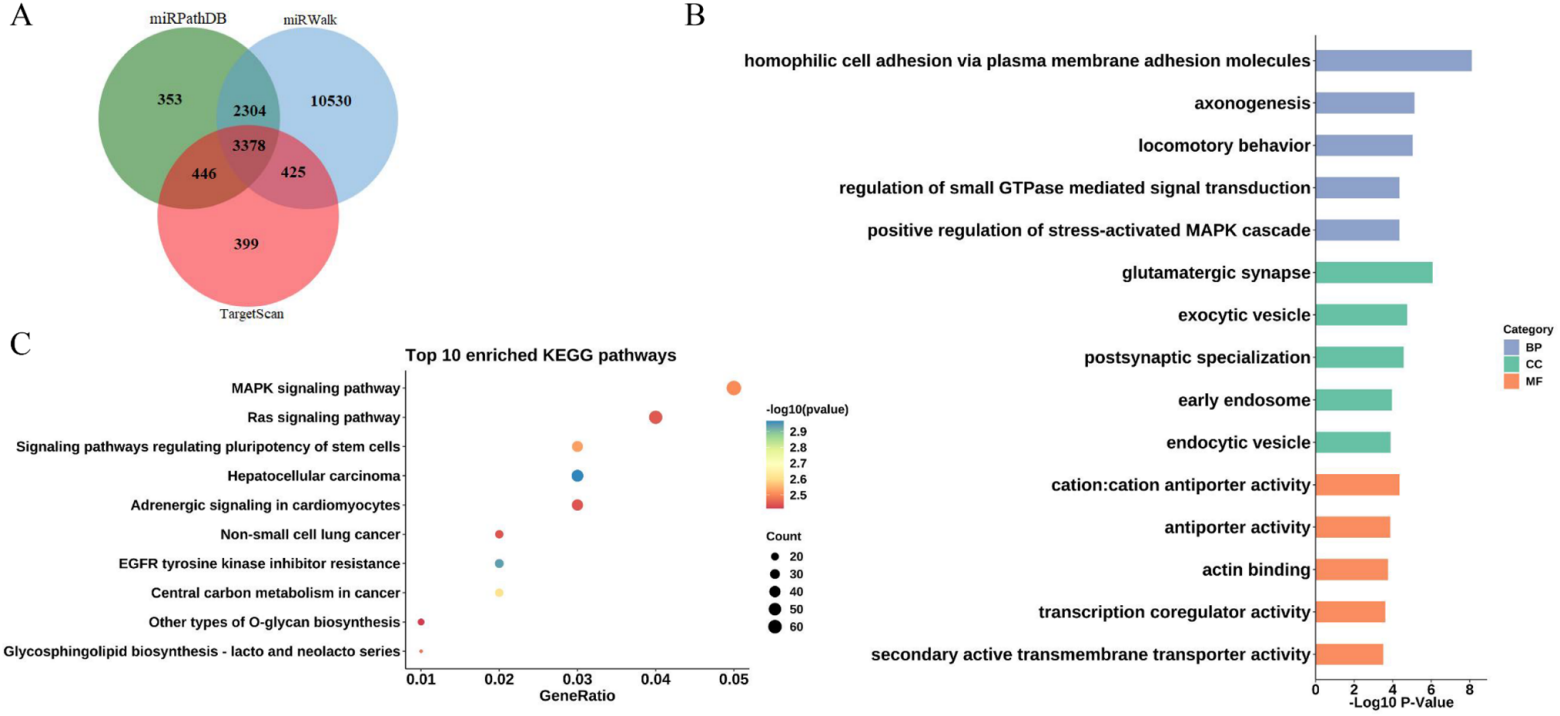
Venn plot of predicted target genes for has-miR-3665 (**A**); GO (**B**) and KEGG (**C**) pathway enrichment analysis of the target genes

A total of 17,835 target genes of hsa-miR-3665 were predicted by miRPathDB, miRWalk and TargetScan. And 3378 target genes were obtained from the intersection of three software predictions (Fig. 2A).

For the 3378 target genes of hsa-miR-3665, GO term enrichment analysis was performed. The top 5 enriched GO terms from three categories are shown in Fig. 2B. For biological processes, regulation of small GTPase-mediated signal transduction (GO:0051056) and positive regulation of stress-activated MAPK cascade (GO:0032874) were significantly enriched. For cellular components, endocytic vesicle (GO:0030139) and early endosome (GO:0005769) were significantly enriched. For molecular functions, antiporter activity (GO:0015297) and actin-binding (GO:0003779) were significantly enriched. When performing the KEGG pathway enrichment analysis, 48 signaling pathways were significantly enriched with the criteria of *P* < 0.05. Furthermore, MAPK signaling pathway, RAS signaling pathway and signaling pathways regulating the pluripotency of stem cells were significantly enriched (Fig. 2C).

### The association between hsa-miR-3665 expression and clinical features

The MS-HRM assay of hsa-miR-3665 promoter methylation in ESCC tumour tissue was analysed by using standard dilutions including 0, 25, 50, 75, and 100% methylation controls (Fig. S1). Hsa-miR-3665 was successfully amplified from all of the ESCC tumour tissue samples (Fig. S2) and further detected according to its methylation levels. Two primer pairs were designed according to the location of the CpG islands (Sangon, Shanghai, China). Based on the promoter sequence of hsa-miR-3665, two pairs of methylation primers were designed (Table S2). The location NC_000013 Chr13:77,698,530–77698738 is named hsa-miR-3665-1, and the location NC_000013 Chr13:77,698,883–77,699,056 is named hsa-miR-3665-2. According to the 50% methylation levels of hsa-miR-3665, 145 ESCC patients were divided into two groups. The correlation coefficient between the expression level and promoter methylation status of hsa-miR-3665 in 145 ESCC samples was 0.646 (*P* < 0.05). As shown in Table 3, we also performed comparisons of the hsa-miR-3665 methylation levels with regard to clinical factors. The hsa-miR-3665-1 methylation levels did not significantly correlate with clinical factors (*P* > 0.05). However, there might be a significant association between hsa-miR-3665-2 methylation levels and TNM stage (*P* = 0.038). To investigate this relationship, a multivariate binary logistic regression analysis was performed. The findings indicated that there was no significant association between the methylation levels of hsa-miR-3665-2 and TNM stage (*OR* = 0.32, 95% *CI*: 0.09 ~ 1.15, Table S6).

**Table 3:** The association between hsa-miR-3665 methylation levels and clinical factors

Variables	hsa-miR-3665-1				hsa-miR-3665-2			
	Low	High	*χ*^*2*^	*P*	Low	High	*χ*^*2*^	*P*
Sex			0.119	0.730			0.437	0.508
Man	14 (73.7)	101 (80.2)			22 (75.9)	79 (81.4)		
Female	5 (26.3)	25 (19.8)			7 (24.1)	18 (18.6)		
Age(years)			0.202	0.653			1.197	0.274
≤ 60	8 (42.1)	60 (47.2)			11 (37.9)	48 (49.5)		
> 60	11 (57.9)	66 (52.4)			18 (62.1)	49 (50.5)		
BMI			1.162	0.559			0.126	0.939
< 18.5	6 (31.6)	26 (20.6)			7 (24.1)	21 (21.6)		
18.5 ~ 24.9	10 (52.6)	73 (57.9)			16 (55.2)	57 (58.8)		
> 25	3 (15.8)	27 (21.4)			6 (20.7)	19 (19.6)		
Tumor location			0.893	0.345			0.648	0.421
Upper/middle	15 (78.9)	86 (68.3)			22 (75.9)	66 (68.0)		
Distal	4 (21.1)	40 (31.7)			7 (24.1)	31 (32.0)		
TNM stage			2.528	0.112			4.308	0.038
I-II	7 (36.8)	71 (56.3)			11 (37.9)	58 (59.8)		
III-IV	12 (63.2)	55 (43.7)			18 (62.1)	39 (40.2)		
Anastomotic fistula			1.815	0.178			0.327	0.568
No	16 (84.2)	120 (95.2)			26 (89.7)	92 (94.8)		
Yes	3 (15.8)	6 (4.8)			3 (10.3)	5 (5.2)		
Postoperative infection			0.007	0.931			0.000	1.000
No	17 (89.5)	108 (85.7)			25 (86.2)	83 (85.6)		
Yes	2 (10.5)	18 (14.3)			4 (13.8)	14 (14.4)		
Invades nerves or vessels			1.255	0.263			1.325	0.250
No	8 (57.1)	32 (41.0)			11 (52.4)	20 (37.7)		
Yes	6 (42.9)	46 (36.5)			10 (47.6)	33 (62.3)		

### Hsa-miR-3665 promoter methylation level might serve as a novel prognostic biomarker in ESCC

The median overall survival time was 48.2 months, and the 1-year, 3-year and 5-year survival rates were 82.4%, 61.2%, and 51.4%, respectively. The Kaplan–Meier method and log-rank test showed no significant difference (for hsa-miR-3665-1, *P* = 0.291; for hsa-miR-3665–2, *P* = 0.657, Fig. S3) in survival between the two groups.

Cox regression was performed to evaluate the prognostic function of the hsa-miR-3665 promoter methylation level in ESCC. The results showed that patients with a high methylation level of hsa-miR-3665-1 had significantly worse survival *(HR* = 3.89, 95% *CI* 1.11 ~ 13.55, Table 4). However, there was no association between the hsa-miR-3665-2 methylation level and OS (*HR* = 1.27, 95% *CI* 0.54 ~ 3.00, Table 4).

**Table 4:** Univariate and multivariate analysis of overall survival in ESCC

Variables	Univariate		Multivariate*		Univariate		Multivariate*	
	*HR*	95% *CI*	*HR*	95% *CI*	*HR*	95% *CI*	*HR*	95%* CI*
Sex								
Men	1.00		1.00		1.00		1.00	
Female	0.65	0.31 ~ 1.37	0.52	0.21 ~ 1.29	0.65	0.31 ~ 1.37	0.68	0.26 ~ 1.81
Age(years)								
≤ 60	1.00		1.00		1.00		1.00	
> 60	1.24	0.73 ~ 2.11	1.37	0.65 ~ 2.92	1.24	0.73 ~ 2.11	1.03	0.45 ~ 2.37
BMI								
< 18.5	1.00		1.00		1.00		1.00	
18.5 ~ 24.9	0.96	0.50 ~ 1.84	0.79	0.29 ~ 2.14	0.96	0.50 ~ 1.84	0.65	0.22 ~ 1.86
> 25	0.52	0.21 ~ 1.26	0.37	0.10 ~ 1.36	0.52	0.21 ~ 1.26	0.31	0.08 ~ 1.25
Tumor location								
Upper/middle	1.00		1.00		1.00		1.00	
Distal	0.48	0.25 ~ 0.95	0.25	0.10 ~ 0.65	0.48	0.25 ~ 0.95	0.20	0.07 ~ 0.62
TNM stage								
I-II	1.00		1.00		1.00		1.00	
III-IV	3.21	1.83 ~ 5.64	3.49	1.50 ~ 8.12	3.21	1.83 ~ 5.64	3.12	1.24 ~ 7.81
Anastomotic fistula								
No	1.00		1.00		1.00		1.00	
Yes	1.53	0.55 ~ 4.25	0.66	0.22 ~ 2.03	1.53	0.55 ~ 4.25	0.67	0.20 ~ 2.19
Postoperative infection								
No	1.00		1.00		1.00		1.00	
Yes	0.81	0.38 ~ 1.71	0.45	0.17 ~ 1.21	0.81	0.38 ~ 1.71	0.50	0.17 ~ 1.43
Invades nerves or vessels								
No	1.00		1.00		1.00		1.00	
Yes	1.94	0.97 ~ 3.86	2.00	0.92 ~ 4.34	1.94	0.97 ~ 3.86	2.14	0.95 ~ 4.83
hsa-mir-3665-1								
≤ 50	1.00		1.00					
> 50	1.63	0.65 ~ 4.10	3.89	1.11 ~ 13.55				
hsa-mir-3665-2								
≤ 50					1.00		1.00	
> 50					0.87	0.46 ~ 1.63	1.27	0.54 ~ 3.00

*Adjusted for sex, age, BMI, tumor location, TNM stage, anastomotic fistula, postoperative infection, invades nerves or vessels, hsa-mir-3665-1/hsa-mir-3665-2

**Fig. 3:**
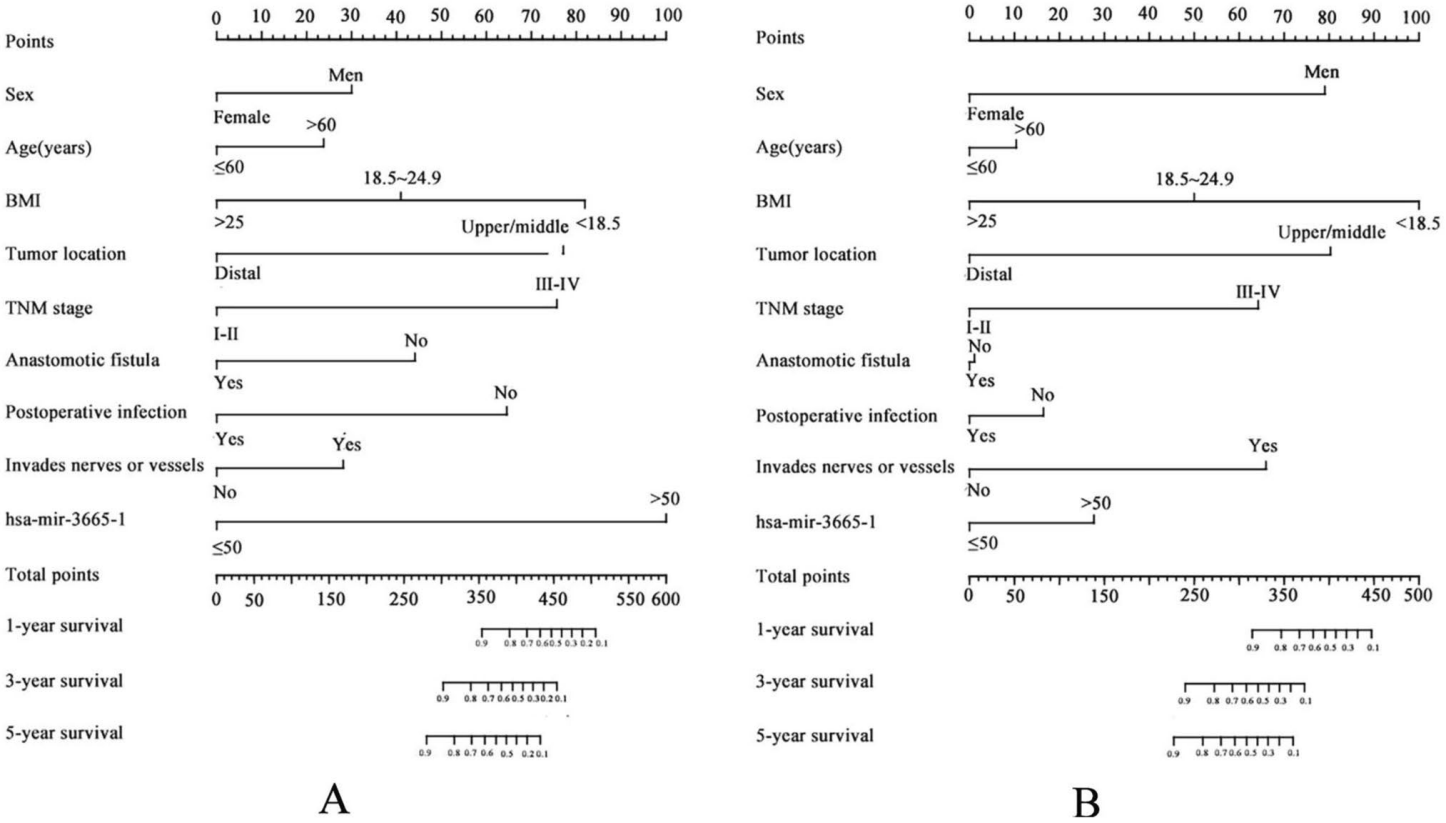
Nomogram predicting the hsa-miR-3665–1 methylation level and clinical factors for patients with ESCC in the training (**A**) and validation sets (**B**)

Moreover, a nomogram incorporating the hsa-miR-3665-1 methylation level and clinical factors was constructed to predict OS in ESCC patients (Fig. 3). The predictive model was virtually presented in the form of a nomogram (Fig. 3) in the training test and was validated with the C-indices of the novel nomogram being 0.748 and 0.751, respectively, reflecting the good discrimination ability of the model. The calibration plot demonstrated that the predicted overall survival probabilities corresponded closely with the observed probabilities (Fig. S4).

## Discussion

MiRNAs are currently implicated in various biological processes [23]. The identification of miRNA promoter methylation will not only enhance our understanding of their role in regulating miRNA expression but also provide prognostic biomarkers for different cancers. In our study, we found that ESCC with a high methylation level of hsa-miR-3665 had a significantly worse prognosis, suggesting that aberrant promoter methylation of hsa-miR-3665 plays a crucial role in ESCC.

Many potential cancer biomarkers for diagnosis and prognosis have been proposed based on miRNA expression levels [24]. However, the methylation state of miRNAs might potentially alter their stability and target recognition, and it might be better than miRNA as a biomarker [25]. Accumulating evidence has indicated that miRNA promoter methylation is related to the progression of cancers and may serve as a potential candidate for predicting the progression of different types of cancers [26, 27]. Vera et al. found that the methylation status of the miR-7 promoter was associated with poorer prognosis in ovarian cancer patients [28]. The hypomethylation of the chromosome 19 microRNA cluster promoter has been declared to be significant for the prognosis evaluation of hepatocellular carcinoma [26]. Meanwhile, Tian et al. [12] showed that the overall survival of ESCC patients with promoter hypermethylation of miR-124-1 (*HR* = 3.245) or miR-124-3 (*HR* = 3.539) was significantly shorter than other ESCC patients and Liu et al. [16] revealed the promoter hypermethylation of miR-203a (*HR* = 1.794) were independently associated with survival of ESCC patients by cox multivariate analysis. In this study, we screened miRNAs with CpG islands from the human miRNA database, which involves a comprehensive assessment of miRNAs. By comprehensive screening of human miRNA promoter methylation, our results showed for the first time that hsa-miR-3665 was epigenetically regulated in ESCC cells, and hypermethylation of the hsa-miR-3665 promoter was associated with a poor prognosis in ESCC, which suggested that it might be an important biomarker for ESCC prognosis.

Promoter hypermethylation of hsa-miR-3665 in ESCC includes hsa-miR-3665-1 and hsa-miR-3665-2. Initial chi-square analysis suggested a potential association between hsa-miR-3665-2 and the TNM stage. However, subsequent multivariate logistic regression analysis, accounting for potential confounding factors, revealed that this association lacked statistical significance. This suggests that the noted correlation might be influenced by confounding effects. Interestingly, after adjusting for covariates, including the TNM stage, multivariate Cox regression analysis demonstrated that the methylation status of hsa-miR-3665-1 is significantly associated with poor prognosis in ESCC. This contrasts with hsa-miR-3665-2, whose promoter region contains fewer CpG sites. This structural difference may explain why the methylation status of hsa-miR-3665-2 appears unrelated to ESCC prognosis.

Although studies have indicated that hsa-miR-3665 dysregulation is closely related to various cancers, the regulatory mechanism and function of hsa-miR-3665 remain elusive. Kilpinen et al. [29] pointed out that aberrant hsa-miR-3665 expression was involved in immune regulation and the cell cycle, and was associated with age-related bone marrow mesenchymal stromal cells. To further explore its function, the target genes of hsa-miR-3665 identified in this study underwent GO and KEGG pathway enrichment analysis. GO enrichment analysis revealed functions in protein transport, cell adhesion, transcription regulator activity, and signal transduction. KEGG pathway analyses revealed that these target genes participate in the regulation of MAPK signaling pathway, RAS signaling pathway and signaling pathways regulating pluripotency of stem cells. The MAPK and RAS signaling pathway play an important role in tumorigenesis and progression by activating related kinases to regulate cell growth, differentiation, proliferation, apoptosis and migration functions [30]. Our results showed that hsa-miR-3665 promoter methylation might affect cell progression in ESCC through MAPK and RAS signaling pathway to associate with the prognosis of ESCC patients. This also implies that hsa-miR-3665 promoter methylation can be used to predict the prognosis of ESCC patients. Nomograms, which combine various clinical factors to predict survival, have been considered useful tools in ESCC [31]. Nevertheless, few studies have analysed the relationship between miRNA promoter methylation and ESCC prognosis by using nomograms. We showed that by incorporating the hsa-miR-3665 methylation level and clinical factors, the nomogram presented good performance for training and validation tests, with C-indices of 0.748 and 0.751, suggesting a better ability to predict the ESCC outcome.

There are several limitations of our study. First, although we validated our results in ESCC tissues, those results were not further validated by experiments such as functional experiments in vitro. It is important to research the potential regulatory mechanisms of hsa-miR-3665 promoter methylation in the progression of ESCC in vitro. In addition, further studies are needed to validate our results with larger samples.

## Conclusions

In summary, we found that a high hsa-miR-3665 promoter methylation level was associated with worse progression in ESCC. Our results suggest that hsa-miR-3665 promoter methylation may be a potential biomarker for predicting the progression of ESCC.

## Supplementary Information

Below is the link to the electronic supplementary material.

Supplementary file1 (PDF 188 KB)

Supplementary file2 (PDF 175 KB)

Supplementary file3 (PDF 255 KB)

Supplementary file4 (PDF 115 KB)

Supplementary file5 (DOCX 36 KB)

## Data Availability

The datasets used and/or analyzed during the current study are available from the corresponding author upon reasonable request.

## Abbreviations

ESCC: Esophageal squamous cell carcinoma
EAC: Esophageal adenocarcinoma
EBI: European Bioinformatics Institute
TNM: Tumor node metastasis
DAC: 5-Aza-2ʹ -deoxycytidine
RQ: Relative quantification
qPCR: Quantitative real-time PCR
MS-HRM: Methylation-specific high resolution melting
GO: Gene ontology
KEGG: Kyoto Encyclopedia of Genes and Genomes
OS: Overall survival
HR: Hazard ratio
95% CI: 95% Confidence interval
C-index: Concordance index
