# Supplementary figures and images for: Discovery and validation of Hsa-microRNA-3665 promoter methylation as a potential biomarker for the prognosis of esophageal squaous cell carcinoma
Source: Int J Clin Oncol. 2024 Dec 4;30(2):309–19. doi: 10.1007/s10147-024-02656-3 (PMC11785691; doi:10.1007/s10147-024-02656-3)

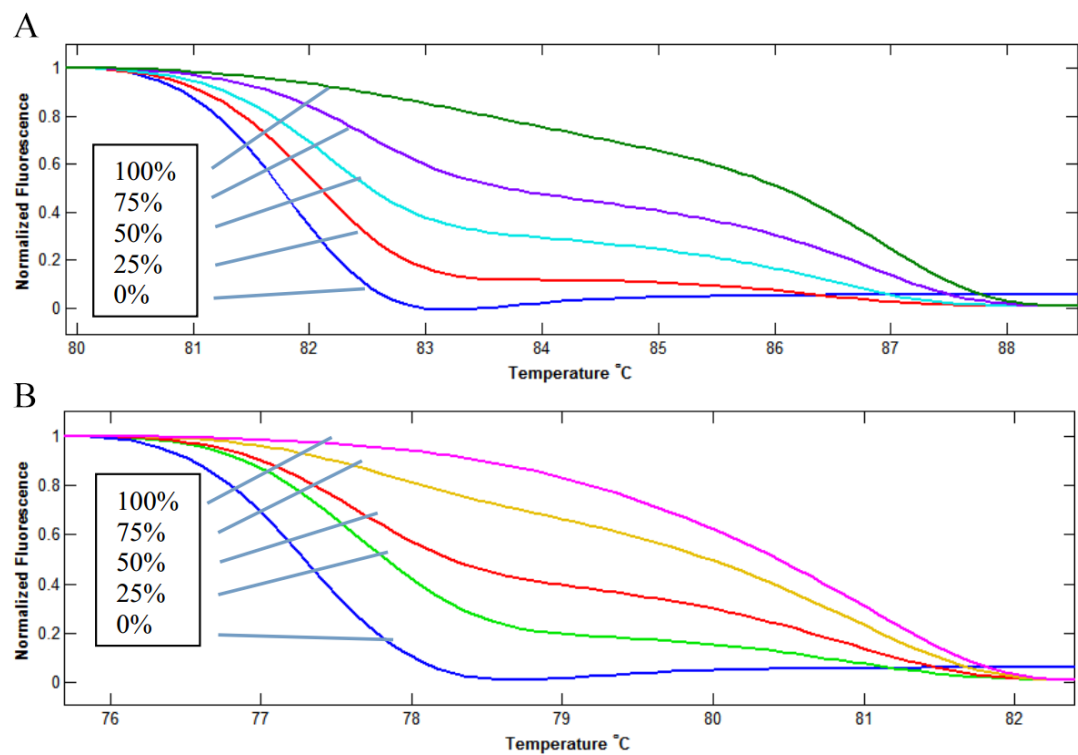

**Figure S1.** The standard serial dilution of has-miR-3665-1 (A), and has-miR-3665-2 (B).

Supplement: Supplementary file 1 — Supplementary file1 (PDF 188 KB) [file 10147_2024_2656_MOESM1_ESM.pdf]

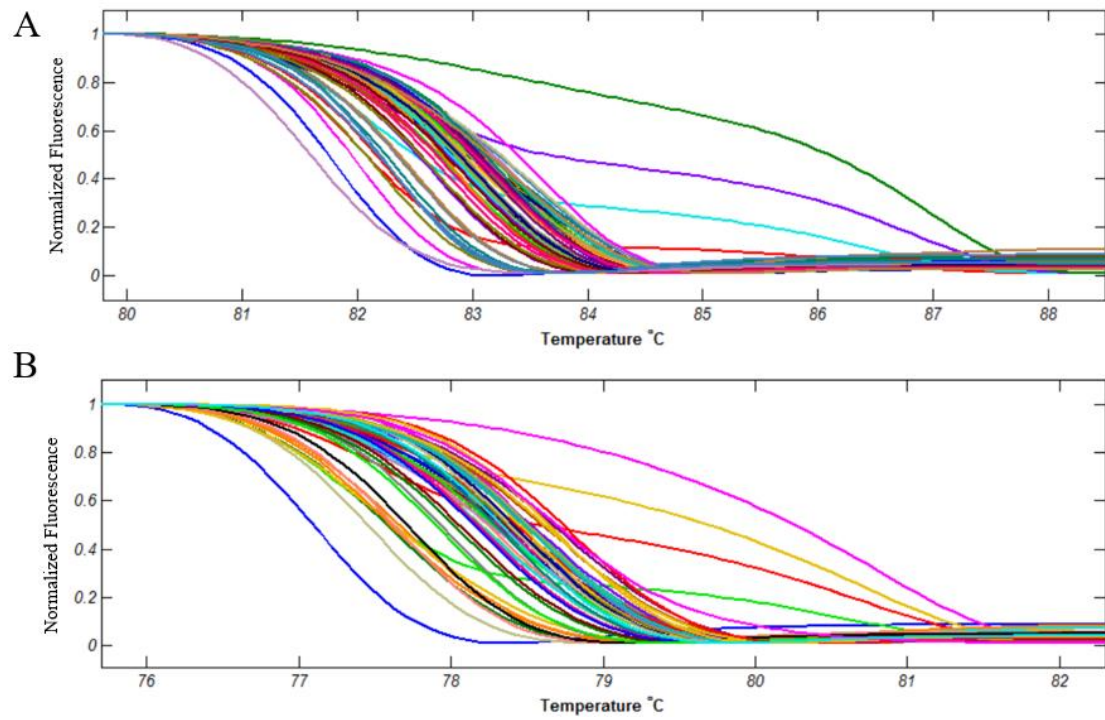

**Figure S2.** The normalized melting curve of hsa-miR-3665(1)(A) and hsa-miR-3665(2) (B) in ESCC tissue.

Supplement: Supplementary file 2 — Supplementary file2 (PDF 175 KB) [file 10147_2024_2656_MOESM2_ESM.pdf]
