# Supplementary material for: Discovery and validation of Hsa-microRNA-3665 promoter methylation as a potential biomarker for the prognosis of esophageal squaous cell carcinoma
Source: Int J Clin Oncol. 2024 Dec 4;30(2):309–19. doi: 10.1007/s10147-024-02656-3 (PMC11785691; doi:10.1007/s10147-024-02656-3)
Supplement: Supplementary file 3 — Supplementary file3 (PDF 255 KB) [file 10147_2024_2656_MOESM3_ESM.pdf]

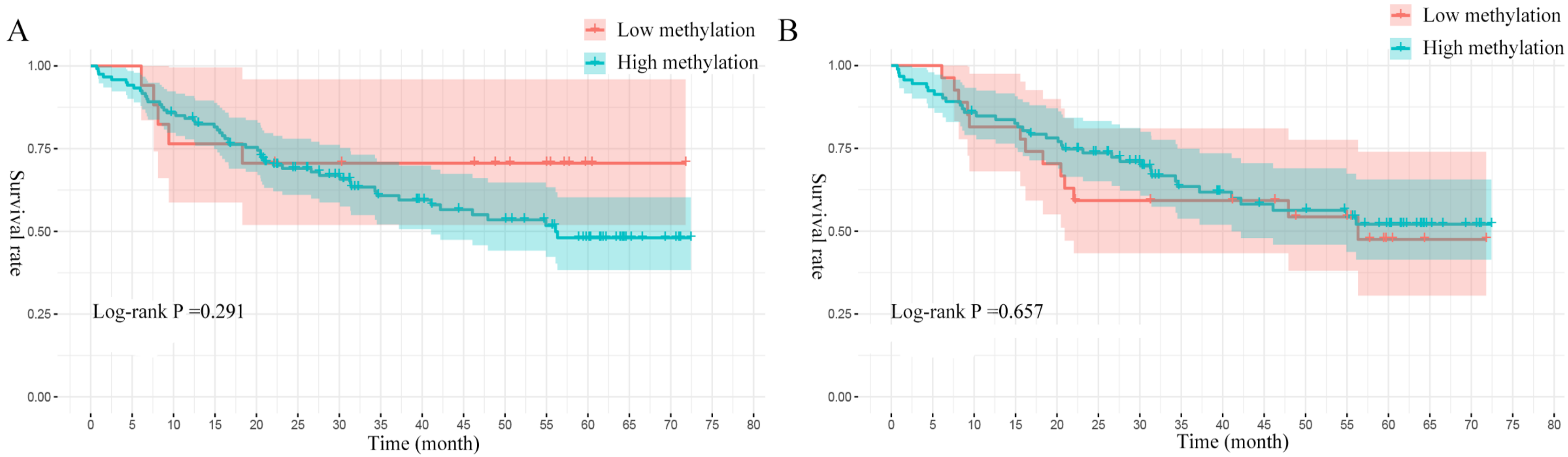

Figure S3. Kaplan-Meier survival curves showing the relationship between high or low methylation level of hsa-miR-3665-1(A), hsa-miR-3665-2 (B) with the overall survival of ESCC.
