# Supplementary material for: Discovery and validation of Hsa-microRNA-3665 promoter methylation as a potential biomarker for the prognosis of esophageal squaous cell carcinoma
Source: Int J Clin Oncol. 2024 Dec 4;30(2):309–19. doi: 10.1007/s10147-024-02656-3 (PMC11785691; doi:10.1007/s10147-024-02656-3)
Supplement: Supplementary file 4 — Supplementary file4 (PDF 115 KB) [file 10147_2024_2656_MOESM4_ESM.pdf]

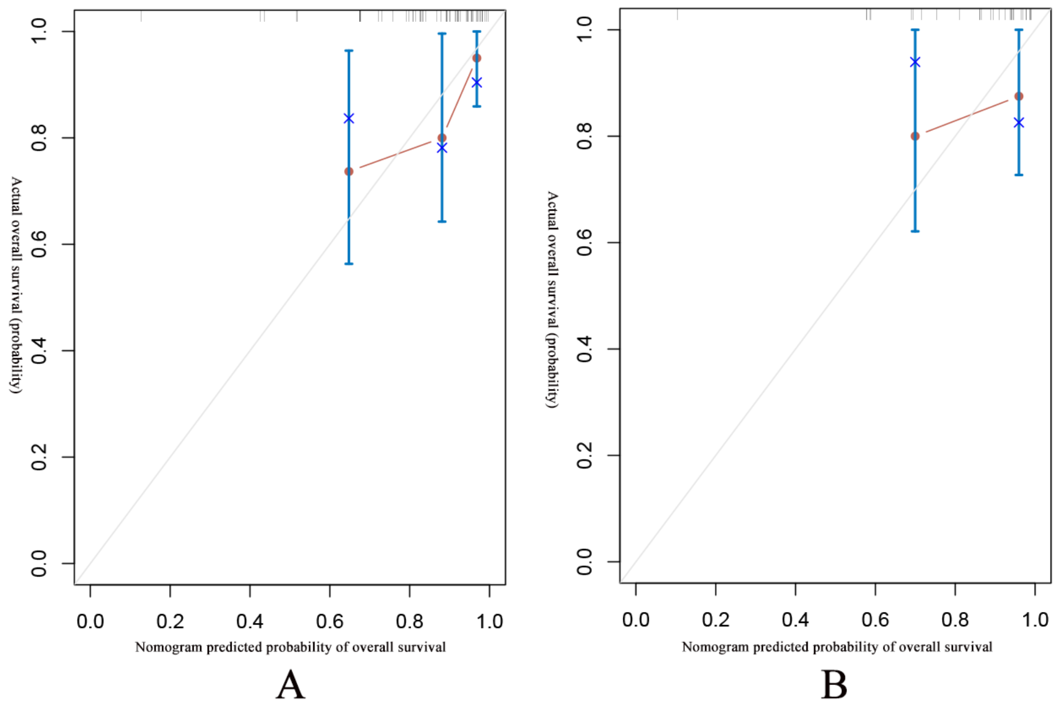

Figure S4. The calibration curve for predicting patient survival in the training (A) and validation sets (B).
