# Supplementary material for: Discovery and validation of Hsa-microRNA-3665 promoter methylation as a potential biomarker for the prognosis of esophageal squaous cell carcinoma
Source: Int J Clin Oncol. 2024 Dec 4;30(2):309–19. doi: 10.1007/s10147-024-02656-3 (PMC11785691; doi:10.1007/s10147-024-02656-3)
Supplement: Supplementary file 5 — Supplementary file5 (DOCX 36 KB) [file 10147_2024_2656_MOESM5_ESM.docx]

**Supplement table 1.** The premier sequence of miProfile^TM^ miRNAs qPCR

| No. | miRNAs | Gene sequence number | Premier identification code |
| --- | --- | --- | --- |
| 1 | hsa-miR-203 | MIMAT0000264 | hsmq-0212 |
| 2 | hsa-miR-210 | MIMAT0000267 | hsmq-0058 |
| 3 | hsa-miR-212-5p | MIMAT0022695 | hsmq-1855 |
| 4 | hsa-let-7i-5p | MIMAT0000415 | hsmq-0586 |
| 5 | hsa-miR-124-5p | MIMAT0004591 | hsmq-0763 |
| 6 | hsa-miR-152 | MIMAT0000438 | hsmq-0074 |
| 7 | hsa-miR-9-5p | MIMAT0000441 | hsmq-0049 |
| 8 | hsa-miR-193a-5p | MIMAT0004614 | hsmq-0552 |
| 9 | hsa-miR-378a-5p | MIMAT0000731 | hsmq-0809 |
| 10 | hsa-miR-596 | MIMAT0003264 | hsmq-0564 |
| 11 | hsa-miR-611 | MIMAT0003279 | hsmq-0566 |
| 12 | hsa-miR-636 | MIMAT0003306 | hsmq-0579 |
| 13 | hsa-miR-638 | MIMAT0003308 | hsmq-0133 |
| 14 | hsa-miR-663a | MIMAT0003326 | hsmq-0560 |
| 15 | hsa-miR-760 | MIMAT0004957 | hsmq-0519 |
| 16 | hsa-miR-935 | MIMAT0004978 | hsmq-0554 |
| 17 | hsa-miR-941 | MIMAT0004984 | hsmq-0565 |
| 18 | hsa-miR-1181 | MIMAT0005826 | hsmq-0008 |
| 19 | hsa-miR-1225-5p | MIMAT0005572 | hsmq-0028 |
| 20 | hsa-miR-1237 | MIMAT0005592 | hsmq-0504 |
| 21 | hsa-miR-1247-5p | MIMAT0005899 | hsmq-0570 |
| 22 | hsa-miR-1258 | MIMAT0005909 | hsmq-0210 |
| 23 | hsa-miR-1281 | MIMAT0005939 | hsmq-0356 |
| 24 | hsa-miR-1292 | MIMAT0005943 | hsmq-0490 |
| 25 | hsa-miR-1538 | MIMAT0007400 | hsmq-0676 |
| 26 | hsa-miR-1539 | MIMAT0007401 | hsmq-0677 |
| 27 | hsa-miR-1914-5p | MIMAT0007889 | hsmq-0684 |
| 28 | hsa-miR-2277-5p | MIMAT0017352 | hsmq-1856 |
| 29 | hsa-miR-2682-5p | MIMAT0013517 | hsmq-1243 |
| 30 | hsa-miR-2861 | MIMAT0013802 | hsmq-0849 |
| 31 | hsa-miR-3153 | MIMAT0015026 | hsmq-1026 |
| 32 | hsa-miR-3178 | MIMAT0015055 | hsmq-1045 |
| 33 | hsa-miR-3181 | MIMAT0015061 | hsmq-0875 |
| 34 | hsa-miR-3187-5p | MIMAT0019216 | hsmq-1857 |
| 35 | hsa-miR-3188 | MIMAT0015070 | hsmq-1049 |
| 36 | hsa-miR-3190-5p | MIMAT0015073 | hsmq-1051 |
| 37 | hsa-miR-3195 | MIMAT0015079 | hsmq-0883 |
| 38 | hsa-miR-4311 | MIMAT0016863 | hsmq-1067 |
| 39 | hsa-miR-4281 | MIMAT0016907 | hsmq-1085 |
| 40 | hsa-miR-3613-5p | MIMAT0017990 | hsmq-1112 |
| 41 | hsa-miR-3615 | MIMAT0017994 | hsmq-1116 |
| 42 | hsa-miR-3621 | MIMAT0018002 | hsmq-1124 |
| 43 | hsa-miR-3652 | MIMAT0018072 | hsmq-1136 |
| 44 | hsa-miR-3655 | MIMAT0018075 | hsmq-1139 |
| 45 | hsa-miR-3661 | MIMAT0018082 | hsmq-1145 |
| 46 | hsa-miR-3663-5p | MIMAT0018084 | hsmq-1147 |
| 47 | hsa-miR-3665 | MIMAT0018087 | hsmq-1150 |
| 48 | hsa-miR-3675-5p | MIMAT0018098 | hsmq-1161 |
| 49 | hsa-miR-3678-5p | MIMAT0018102 | hsmq-1165 |
| 50 | hsa-miR-3687 | MIMAT0018115 | hsmq-1178 |
| 51 | hsa-miR-3939 | MIMAT0018355 | hsmq-1222 |
| 52 | hsa-miR-4449 | MIMAT0018968 | hsmq-1288 |
| 53 | hsa-miR-4453 | MIMAT0018975 | hsmq-1294 |
| 54 | hsa-miR-4469 | MIMAT0018996 | hsmq-1238 |
| 55 | hsa-miR-4470 | MIMAT0018997 | hsmq-1315 |
| 56 | hsa-miR-4479 | MIMAT0019011 | hsmq-1329 |
| 57 | hsa-miR-4482-5p | MIMAT0019016 | hsmq-1333 |
| 58 | hsa-miR-4515 | MIMAT0019052 | hsmq-1369 |
| 59 | hsa-miR-4522 | MIMAT0019060 | hsmq-1378 |
| 60 | hsa-miR-4530 | MIMAT0019069 | hsmq-1388 |
| 61 | hsa-miR-4634 | MIMAT0019691 | hsmq-1413 |
| 62 | hsa-miR-4638-5p | MIMAT0019695 | hsmq-1417 |
| 63 | hsa-miR-4651 | MIMAT0019715 | hsmq-1437 |
| 64 | hsa-miR-4664-5p | MIMAT0019737 | hsmq-1459 |
| 65 | hsa-miR-4665-5p | MIMAT0019739 | hsmq-1461 |
| 66 | hsa-miR-2964a-5p | MIMAT0019747 | hsmq-1718 |
| 67 | hsa-miR-4674 | MIMAT0019756 | hsmq-1477 |
| 68 | hsa-miR-4683 | MIMAT0019768 | hsmq-1489 |
| 69 | hsa-miR-4687-5p | MIMAT0019774 | hsmq-1495 |
| 70 | hsa-miR-4734 | MIMAT0019859 | hsmq-1580 |
| 71 | hsa-miR-4738-5p | MIMAT0019866 | hsmq-1587 |
| 72 | hsa-miR-4757-5p | MIMAT0019901 | hsmq-1622 |
| 73 | hsa-miR-4787-5p | MIMAT0019956 | hsmq-1677 |
| 74 | hsa-miR-4792 | MIMAT0019964 | hsmq-1685 |
| 75 | hsa-miR-191-5p | MIMAT0000440 | hsmq-0079 |
| 76 | hsa-miR-34c-5p | MIMAT0000686 | hsmq-0123 |
| 77 | hsa-miR-375 | MIMAT0000728 | hsmq-0544 |
| 78 | hsa-miR-92b-5p | MIMAT0004792 | hsmq-0837 |
| 79 | hsa-miR-3180-5p | MIMAT0015057 | hsmq-1046 |
| 80 | hsa-miR-1302 | MIMAT0005890 | hsmq-0238 |
| 81 | hsa-miR-4523 | MIMAT0019061 | hsmq-1379 |
| 82 | hsa-miR-345-5p | MIMAT0000772 | hsmq-0448 |
| 83 | hsa-miR-564 | MIMAT0003228 | hsmq-0527 |
| 84 | hsa-miR-4497 | MIMAT0019032 | hsmq-1349 |
| 85 | hsa-miR-4519 | MIMAT0019056 | hsmq-1373 |
| 86 | hsa-miR-4741 | MIMAT0019871 | hsmq-1592 |
| 87 | hsa-miR-4785 | MIMAT0019949 | hsmq-1670 |
| 88 | hsa-miR-572 | MIMAT0003237 | hsmq-0114 |
| 89 | hsnoRNA U6 ^#^ | NR_002752 | RNU6B |
| 90 | hsnoRNA U44 ^#^ | NR_002750 | SNORD44 |
| 91 | hsnoRNA U48 ^#^ | NR_002745.1 | SNORD48 |
| 92 | hsnoRNA U47 ^#^ | NR_002746 | SNORD47 |
| 93 | RTC* | / | / |
| 94 | PPC** | / | / |
| 95 | NTC*** | NR_002752 | RNU6B |

#: U6, U44, U48 and U47 as internal reference genes; *RTC: Reverse transcription control, this well cross-linked with primers for exogenous standard RNA reverse transcription products; **PPC: Positive PCR control, this well cross-linked with the corresponding primers, without adding their own template, plus positive control cDNA; ***NTC: No Template Control, this well cross-linked with primers for the internal reference gene U6, and the reaction was performed with water instead of template.

**Supplement table 2.** The premier sequence of has-miR-3665

| miRNA | Sequence | Forward | Reverse | Location (NCBI,GenBank ,2014) |
| --- | --- | --- | --- | --- |
| hsa-miR-3655-1 | NC_000013.11 | GTTTTTGTTTTTATGAGAAGTTTTAT | ACTACACCTAATACTACCTAACTCC | NC_000013 Chr13:77698530-77698738 |
| hsa-miR-3655-2 | NC_000013.11 | TTTTTATAAAGAGAGATTTATTATTG | TACTACTCTTTTCCCCAATATTTC | NC_000013 Chr13:77698883-77699056 |

**Supplement table 3.** Comprehensive screening of eighty-eight miRNA promoter methylation

| Number | miRNAs | GC%* | ObsCpG/ExpCpG* |
| --- | --- | --- | --- |
| 1 | hsa-mir-203 | 75.9 | 0.902 |
| 2 | hsa-mir-210 | 79.2 | 0.852 |
| 3 | hsa-mir-212 | 75.1 | 0.864 |
| 4 | hsa-let-7i | 69.4 | 1.020 |
| 5 | hsa-mir-124-3 | 73.0 | 1.018 |
| 6 | hsa-mir-152 | 72.0 | 0.776 |
| 7 | hsa-mir-9-3 | 65.5 | 0.846 |
| 8 | hsa-mir-193a | 76.8 | 0.894 |
| 9 | hsa-mir-378a | 66.4 | 0.990 |
| 10 | hsa-mir-596 | 62.5 | 0.902 |
| 11 | hsa-mir-611 | 63.0 | 0.782 |
| 12 | hsa-mir-636 | 72.5 | 1.061 |
| 13 | hsa-mir-638 | 68.7 | 0.998 |
| 14 | hsa-mir-663a | 71.7 | 0.897 |
| 15 | hsa-mir-760 | 66.7 | 0.896 |
| 16 | hsa-mir-935 | 70.3 | 0.863 |
| 17 | hsa-mir-941-3 | 66.6 | 0.701 |
| 18 | hsa-mir-1181 | 65.5 | 0.746 |
| 19 | hsa-mir-1225 | 73.0 | 0.792 |
| 20 | hsa-mir-1237 | 68.5 | 0.723 |
| 21 | hsa-mir-1247 | 81.2 | 0.981 |
| 22 | hsa-mir-1258 | 69.5 | 0.832 |
| 23 | hsa-mir-1281 | 74.5 | 0.886 |
| 24 | hsa-mir-1292 | 68.6 | 0.989 |
| 25 | hsa-mir-1538 | 73.3 | 0.807 |
| 26 | hsa-mir-1539 | 65.0 | 0.991 |
| 27 | hsa-mir-1914 | 67.0 | 0.706 |
| 28 | hsa-mir-2277 | 66.9 | 0.841 |
| 29 | hsa-mir-2682 | 63.6 | 0.845 |
| 30 | hsa-mir-2861 | 76.8 | 0.859 |
| 31 | hsa-mir-3153 | 65.4 | 0.881 |
| 32 | hsa-mir-3178 | 69.2 | 0.837 |
| 33 | hsa-mir-3181 | 70.8 | 0.860 |
| 34 | hsa-mir-3187 | 76.3 | 0.941 |
| 35 | hsa-mir-3188 | 76.7 | 0.922 |
| 36 | hsa-mir-3190 | 72.1 | 0.804 |
| 37 | hsa-mir-3195 | 77.7 | 0.782 |
| 38 | hsa-mir-4311 | 64.1 | 0.731 |
| 39 | hsa-mir-4281 | 70.3 | 0.825 |
| 40 | hsa-mir-3613 | 69.0 | 0.800 |
| 41 | hsa-mir-3615 | 67.3 | 0.923 |
| 42 | hsa-mir-3621 | 73.9 | 0.824 |
| 43 | hsa-mir-3652 | 63.9 | 0.816 |
| 44 | hsa-mir-3655 | 61.0 | 0.876 |
| 45 | hsa-mir-3661 | 67.0 | 0.765 |
| 46 | hsa-mir-3663 | 66.1 | 0.725 |
| 47 | hsa-mir-3665 | 72.2 | 0.798 |
| 48 | hsa-mir-3675 | 64.2 | 0.708 |
| 49 | hsa-mir-3678 | 69.4 | 0.839 |
| 50 | hsa-mir-3687 | 74.2 | 1.154 |
| 51 | hsa-mir-3939 | 69.0 | 0.813 |
| 52 | hsa-mir-4449 | 69.9 | 0.803 |
| 53 | hsa-mir-4453 | 72.5 | 0.810 |
| 54 | hsa-mir-4469 | 76.7 | 1.045 |
| 55 | hsa-mir-4470 | 69.2 | 0.895 |
| 56 | hsa-mir-4479 | 70.7 | 1.012 |
| 57 | hsa-mir-4482-1 | 61.4 | 0.778 |
| 58 | hsa-mir-4515 | 70.2 | 0.902 |
| 59 | hsa-mir-4522 | 66.5 | 0.779 |
| 60 | hsa-mir-4530 | 66.7 | 0.714 |
| 61 | hsa-mir-4634 | 63.5 | 0.980 |
| 62 | hsa-mir-4638 | 60.4 | 0.785 |
| 63 | hsa-mir-4651 | 68.8 | 0.815 |
| 64 | hsa-mir-4664 | 77.6 | 0.853 |
| 65 | hsa-mir-4665 | 66.1 | 0.802 |
| 66 | hsa-mir-2964a | 64.1 | 0.968 |
| 67 | hsa-mir-4674 | 70.1 | 0.817 |
| 68 | hsa-mir-4683 | 72.8 | 0.917 |
| 69 | hsa-mir-4687 | 72.6 | 0.812 |
| 70 | hsa-mir-4734 | 67.6 | 1.034 |
| 71 | hsa-mir-4738 | 65.4 | 0.794 |
| 72 | hsa-mir-4757 | 64.0 | 0.815 |
| 73 | hsa-mir-4787 | 73.7 | 0.957 |
| 74 | hsa-mir-4792 | 70.2 | 0.828 |
| 75 | hsa-mir-191 | 67.3 | 0.800 |
| 76 | hsa-mir-34c | 69.5 | 0.856 |
| 77 | hsa-mir-375 | 70.7 | 0.838 |
| 78 | hsa-mir-92b | 65.6 | 0.877 |
| 79 | hsa-mir-3180-2 | 68.8 | 0.801 |
| 80 | hsa-mir-1302-11 | 72.0 | 0.868 |
| 81 | hsa-mir-4523 | 67.8 | 0.841 |
| 82 | hsa-mir-345 | 70.4 | 0.867 |
| 83 | hsa-mir-564 | 65.7 | 0.860 |
| 84 | hsa-mir-4497 | 73.8 | 0.815 |
| 85 | hsa-mir-4519 | 65.3 | 0.840 |
| 86 | hsa-mir-4741 | 65.6 | 0.905 |
| 87 | hsa-mir-4785 | 72.4 | 0.963 |
| 88 | hsa-mir-572 | 64.2 | 1.093 |

* GC% and ObsCpG/ExpCpG data from CpG Island Searcher.

**Supplement table 4.** Relative expression of 15 miRNAs from ten ESCC

| MiRNAs | 1 | 2 | 3 | 4 | 5 | 6 | 7 | 8 | 9 | 10 |
| --- | --- | --- | --- | --- | --- | --- | --- | --- | --- | --- |
| hsa-miR-9-5p | 0.188 | 0.729 | 1.239 | 5.194 | 0.027 | 1.589 | 0.140 | 0.045 | 1.371 | 3.141 |
| hsa-miR-203 | 0.547 | 0.318 | 0.288 | 321.443 | 0.317 | 16.893 | 13.271 | 3.442 | 0.044 | 0.009 |
| hsa-miR-375 | 0.036 | 0.216 | 0.077 | 40.284 | 0.127 | 1.790 | 1.641 | 0.922 | 0.377 | 0.021 |
| hsa-miR-1258 | 0.537 | 2.010 | 0.691 | 2.721 | 0.023 | 1.697 | 0.717 | 0.117 | 3.323 | 1.134 |
| hsa-miR-1914-5p | 1.003 | 5.239 | 0.524 | 2.078 | 0.108 | 0.577 | 0.477 | 0.083 | 1.100 | 1.533 |
| hsa-miR-3665 | 0.019 | 0.144 | 0.356 | 4.438 | 0.019 | 0.536 | 0.131 | 0.073 | 2.333 | 0.414 |
| hsa-miR-4470 | 2.365 | 1.775 | 0.706 | 4.715 | 0.057 | 3.543 | 0.878 | 0.112 | 5.145 | 1.689 |
| hsa-miR-4479 | 0.101 | 1.701 | 0.881 | 5.541 | 0.090 | 1.146 | 0.641 | 0.166 | 3.954 | 1.351 |
| hsa-miR-4530 | 0.112 | 1.745 | 0.731 | 2.950 | 0.091 | 0.906 | 0.189 | 0.057 | 3.302 | 1.206 |
| hsa-miR-4634 | 0.134 | 1.926 | 0.412 | 2.877 | 0.042 | 0.543 | 0.104 | 0.094 | 2.101 | 1.003 |
| hsa-miR-4664-5p | 1.279 | 0.838 | 0.371 | 104.959 | 0.033 | 0.741 | 0.721 | 0.059 | 5.570 | 2.645 |
| hsa-miR-4665-5p | 0.059 | 2.058 | 0.626 | 10.192 | 0.075 | 0.676 | 0.190 | 0.066 | 4.251 | 1.660 |
| hsa-miR-4674 | 0.100 | 2.410 | 0.536 | 11.611 | 0.127 | 2.111 | 0.541 | 0.120 | 8.745 | 1.036 |
| hsa-miR-4687-5p | 0.261 | 0.805 | 1.336 | 0.183 | 0.068 | 7.314 | 0.125 | 0.045 | 5.579 | 0.763 |
| hsa-miR-4734 | 0.182 | 4.779 | 1.025 | 34.313 | 0.388 | 1.107 | 0.384 | 0.046 | 4.045 | 2.836 |

**Supplement table 5.** The correlation coefficient between the expression level and promoter methylation status of hsa-miR-3665 in ten ESCC

| MiRNAs | r | P |
| --- | --- | --- |
| hsa-miR-3665-1 | -0.692 | 0.027 |
| hsa-miR-3665-2 | -0.588 | 0.074 |

**Supplement table 6.** A multivariate binary logistic regression analyses of the association between hsa-miR-3665-2 methylation levels and TNM stage

| Variables* |  | *OR* | 95% *CI* | *P* |
| --- | --- | --- | --- | --- |
| hsa-mir-3665-2 |  |  |  |  |
| ≤50 |  | 1.00 |  |  |
| >50 |  | 0.32 | 0.09~1.15 | 0.082 |

*Adjusted for sex, age, BMI, tumor location, anastomotic fistula, postoperative infection, invades nerves or vessels
